# Separation and purification of plant terpenoids from biotransformation

**DOI:** 10.1002/elsc.202100014

**Published:** 2021-06-10

**Authors:** Linhao Chen, Yaru Pang, Yan Luo, Xu Cheng, Bo Lv, Chun Li

**Affiliations:** ^1^ Key Laboratory of Medical Molecule Science and Pharmaceutics Engineering Ministry of Industry and Information Technology Institute of Biochemical Engineering School of Chemistry and Chemical Engineering Beijing Institute of Technology Beijing P. R. China; ^2^ Key Lab for Industrial Biocatalysis Ministry of Education Department of Chemical Engineering Tsinghua University Beijing P. R. China

**Keywords:** intelligent microbial cell factories, plant terpenoids, purification, separation, synthetic biology

## Abstract

The production of plant terpenoids through biotransformation has undoubtedly become one of the research hotspots, and the continuous upgrading of the corresponding downstream technology is also particularly important. Downstream technology is the indispensable technical channel for the industrialization of plant terpenoids. How to efficiently separate high‐purity products from complex microbial fermentation broths or enzyme‐catalyzed reactions to achieve high separation rates, high returns and environmental friendliness has become the focus of research in recent years. This review mainly introduces the common separation methods of plant terpenoids based on biotransformation from the perspectives of engineering strain construction, unit separation technology, product properties and added value. Then, further attention was paid to the application prospects of intelligent cell factories and control in the separation of plant terpenoids. Finally, some current challenges and prospects are proposed, which provide possible directions and guidance for the separation and purification of terpenoids and even industrialization.

**Abbreviations**
DCS: distributed control systems
GA: glycyrrhetinic acid
GAMG: glycyrrhetinic acid 3‐O‐mono‐β‐D‐glucuronide
GL: glycyrrhizin
GlcA: glucuronic acid
PLC: programmable logic controller
TRPU: temperature‐responsive polyurethane

## 1 INTRODUCTION

**FIGURE 1:**
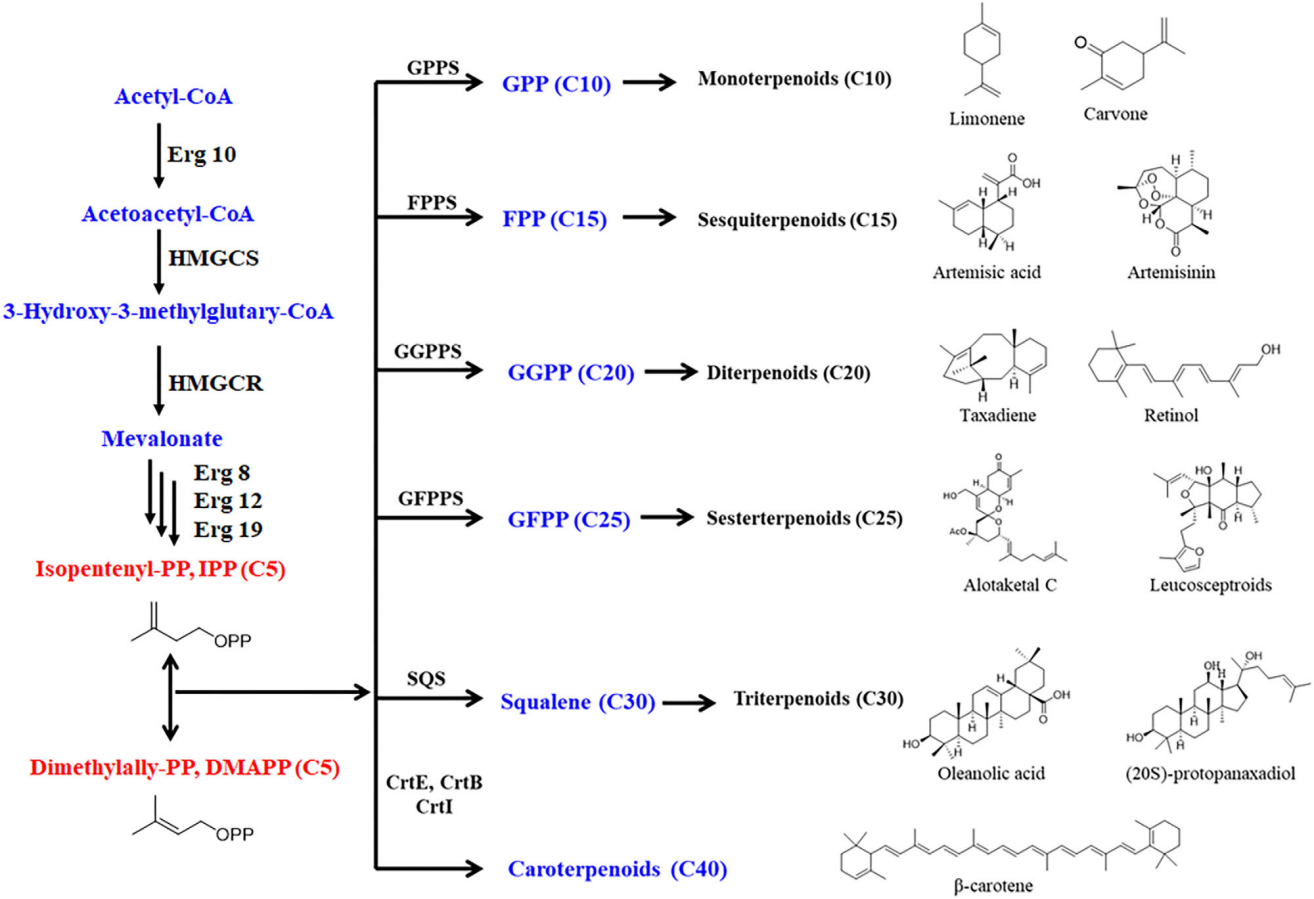
Brief overview and examples of biosynthetic pathways in plant terpenoids. Crt, including carotenoid synthase such as crtE, crtY, crtI, and crtB; DMAPP, dimethylallyl pyrophosphate; GPPS, geranyl pyrophosphate synthase; IPP, isopentenyl pyrophosphate; FPPS, farnesyl‐diphosphate synthase; GGPPS, geranylgeranylpyrophosphate synthase; GFPPS, geranylfranesyl‐PP synthase; SQS, squalene synthase

Terpenoids are a large group of important natural products, which are ubiquitous in plants and a few in animals. At present, more than 80,000 kinds of terpenoids have been discovered [1]. Terpenoids can be classified depending on the number of isoprene units into hemiterpenes (C5), monoterpenoids (C10), sesquiterpenoids (C15), diterpenoids (C20), sesterterpenoids (C25), triterpenoids (C30), and tetraterpenes (C40) (Figure 1). The structural diversity of terpenoids displays various biological activities and high application value in many fields such as medicine, food, cosmetics and biofuels [2, 3, 4] (Table 1). For example, glycyrrhizin (GL) and glycyrrhetinic acid (GA) are typical triterpenoids in the plant licorice, which have antiviral, anti‐inflammatory, anti‐tumor and liver‐protective effects [5].

**FIGURE 2:**
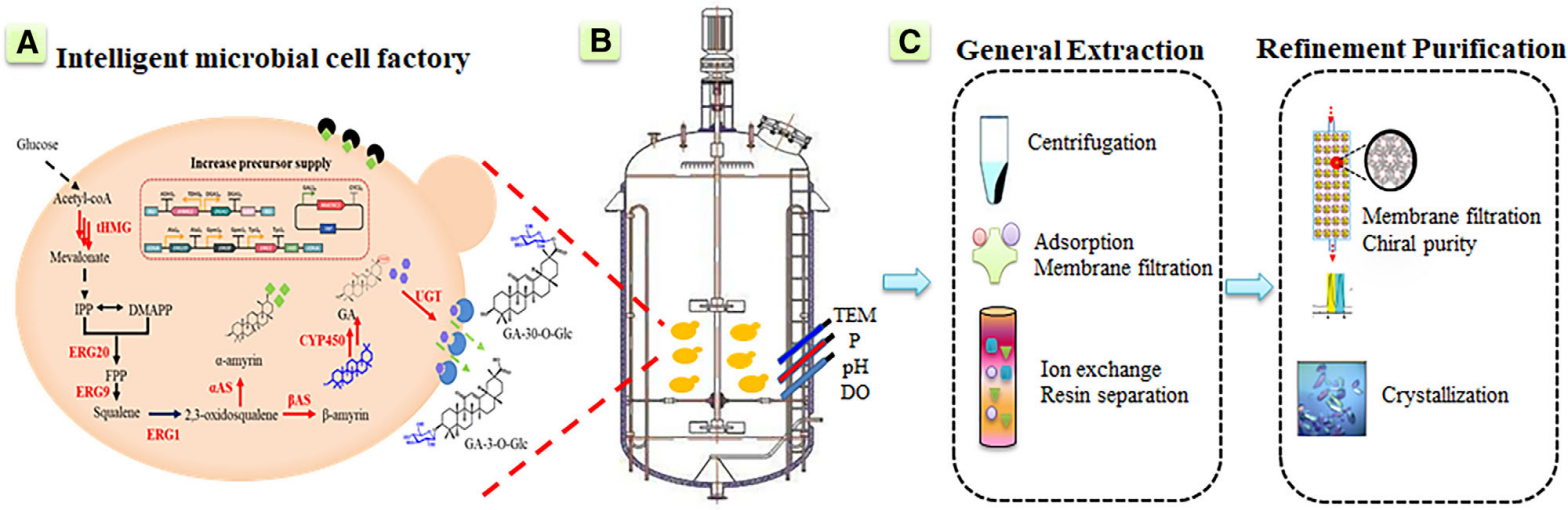
Upstream strain construction strategies to promote downstream separation process. (A) Intelligent microbial cell factory including glycosylation modification, increase the supply of precursors, morphology engineering, transmembrane transport, cell‐free system, inducible cell lysis systems and so on; (B) Intelligent control and parameters monitoring of fermentation system; (C) Preliminary separation and fine separation in the downstream process of terpenoids

**TABLE 1:** Typical application of terpenoids and their saponins

Compound	Types	Function	References
Limonene	Monoterpenoids	Mosquito repellent; antioxidation; antimicrobial packaging	[40, 41, 42]
Artemisinin and its derivatives	Sesquiterpenoids	The best drug to treat resistance to malaria	[43]
Vitamin A	Diterpenoids	Protecting eyesight and preventing eye disease; promoting the growth and development; hair care	[44, 45]
Alotaketal C	Sesterterpenoids	Potential anti‐HIV drug precursors	[46]
Oleanolic acid	Triterpenoids	Inhibiting the growth of liver cancer cells; anti‐inflammatory	[47]
Glycyrrhizin	Triterpenoid saponins	Repressing prostate cancer metastasis; inhibiting osteoarthritis development; naturally‐derived surfactant	[48, 49, 50]

As people pay close attention to health, environment and energy issues, the demand for natural terpenoids is growing rapidly [6, 7, 8]. At present, the industrial production of terpenoids mainly relies on extraction from plants, followed by chemical synthesis. However, these two production methods have many defects. Plant extraction methods usually take up a lot of land resources, and the planting of raw materials is easily affected by weather factors. In addition, the low content of target products in plants and many structural analogs often lead to problems such as low separation and purification efficiency. Terpenoids can also be produced by chemical synthesis, with a large amount of organic reagents and energy‐consumed, or lead to a serious environmental pollution. Fortunately, synthetic biology can provide a sustainable method for the industrial‐scale production of terpenoids [9]. For example, Shukal et al. systematically optimized the auxotrophic *Escherichia coli* through a variety of synthetic biology methods to produce valuable viridiflorol, a sesquiterpene alcohol that is widely used in cosmetics and personal care products, making the yield of viridiflorol reached 25.7 g/L in 2.5 days. A similar method was applied to the production of amorphadiene, the precursor of the antimalarial drug artemisinin, and its yield reached 30 g/L [10].

Moreover, the high separation high and high efficiency high technologies are the most critical challenge in downstream separation. The high concentration of end‐product is benefit to lowering the cost of downstream processing [11]. Usually, downstream separation accounts for a very high proportion of the production cost (even more than 70% of the total cost). Biotransformation also offers a new route to produce and purify terpenoids from certain phytochemical derivatives, such as glycosylation, methylation, acetylation and amidation. Apart from solubleness, centrifugation is being employed extensively due its simplicity and effectiveness. Quercetin, derived from rutin by detachment of glucose and rhamnose molecules, is highly insoluble in water [12]. Biotransformation via methylation could lead to phase separation due to more hydrophobic interaction.

Generally, for the separation and purification of plant terpenoids, we need to pay attention to the following points: (1) How to construct upstream strains to increase yield and facilitate downstream separation; (2) For terpenoids of different properties and added value, how to choose a more effective separation technology to achieve a sustainable process with low cost and high yield; (3) Continuously develop and improve unit separation technology, such as in‐situ production and separation by combining bioreactors; (4) How to couple the intelligent cell factory and control with the efficient production and separation of terpenoids; (5) Moving closer to the goal of cleaner production, etc.

**PRACTICAL APPLICATION**

Terpenoids are a large group of important natural products, and more than 80,000 kinds of terpenoids have been discovered. The structural diversity of terpenoids gives it many different biological activities and is widely used in many fields such as medicine, food, cosmetics and biofuels. For the industrialization of terpenoid products, the downstream separation process is one of the most critical links. In order to overcome the shortcomings of plant extraction and chemical synthesis, combining synthetic biology and downstream processing technology to separate high‐purity terpenoids from fermentation broth or enzyme‐catalyzed reactions has become a research hotspot in recent years. Therefore, this review summarizes the separation and purification strategies of plant terpenoids from the perspectives of the construction of upstream engineering bacteria, common unit separation technology, intelligent cell factories and control, which provide possible directions and guidance for the separation and purification of terpenoids and even industrialization.

In this review, we summarize the isolation and purification strategies of plant terpenoids. First, briefly introduce some methods that facilitate downstream separation from the construction of upstream engineering strain. At the same time, from the perspective of product properties and added value, we focus on describing some effective and common downstream separation techniques in preliminary and fine separation, including extraction, crystallization, membrane separation, chromatography, ion exchange and so on. Then, we mainly take terpenoids as examples to introduce the application prospects of intelligent cell factories and control in separation and purification. Finally, the separation and purification of plant terpenoids are evaluated and prospected based on recent studies.

## 2 ENGINEERED STRAINS FOR DOWNSTREAM SEPARATION

The usual cost in terpenoids production consisted of two parts, upstream and downstream (Figure 2). For industrial production, most researchers pay attention to the yield, stability of the engineered strains in the application process and whether it is beneficial to simplify downstream separation. In recent years, there have been many methods that promote the production of terpenoids and facilitate the downstream separation process, such as glycosylation modification, precursor supply, chassis strain modification [13], cell‐free systems and induced cell lysis system, etc (Table 2).

**TABLE 2:** Developed strain construction strategies for downstream separation

Strategy	How to promote downstream separation	Products	Culture conditions	Result	References
Glycosylation modification	(1) Increase the lipophilicity of the compound by reducing the glycoside in the compound structure and increase its solubility in organic solvents	GAMG	Fed‐batch fermentation in a 150‐L fermentor	25.3 g/L	[16]
	(2) Increase the hydrophilicity of the product by adding glycosides to the compound structure and increase its solubility in the water phase	Glycyrrhetinic acid‐3‐O‐Glc	In vitro biosynthesis	8.2 g/L	[17]
Increase the supply of precursors	Increasing the availability of synthetic precursors to facilitate the production of products has a positive impact on downstream processing	Viridiflorol and amorphadiene	Fed‐batch fermentation	25.7 g/L; 30 g/L	[10]
		(−)‐α‐bisabolol	Fed‐batch fermentation	8.5 g/L	[51]
		Geranyl acetate	Fed‐batch fermentation based on two‐phase system	4.8 g/L	[52]
		Amorpha‐4,11‐diene	Fed‐batch fermentation	40 g/L	[53]
Chassis strain modification	(1) Morphology engineering: By changing the cell shape or the state of organelles to accommodate more intracellular products and it is also conducive to natural settlement or to improve the centrifugal effect	α‐Amyrin	Fed‐batch fermentation	1.1 g/L	[18]
		Squalene	Two‐stage fed‐batch fermentation	11 g/L	[54]
	(2) Lipid engineering: By improving the orientation of the substrate and reducing the toxicity caused by product accumulation, thereby increasing the yield of the product	Lycopene	Fed‐batch fermentation	2.37 g/L	[19]
	(3a) Transmembrane transport: Transfer the substrate into the cell to promote the occurrence of intracellular reactions, thereby increasing the yield;	Hydrocortisone	Shake flasks	1.06 g/L	[20]
	(3b) Transmembrane transport: By modifying the strain to transport intracellular products to the outside of the cell, reducing cell disruption and other operations	Artemisinic acid	In a 1‐L aerated bioreactor	115 mg/L	[55]
Cell‐free system	Reduce centrifugation and cell disruption, etc.	Limonene	Glass vials	15 g/L	[23]
Inducible cell lysis systems	Introducing lysis genes (such as P_BAD_‐AraC system, P_mgt_B promoter or P_R_‐CI857 system [24, 56‐58]) to achieve cell‐induced lysis under specific conditions, simplifying downstream processing procedures	Polyhydroxyalkanoates (PHAs)	Two phases fermentation system	0‐1 g/L	[59]

### 2.1 Glycosylation modification

Structural modification is an effective tool for changing the lipophilicity and hydrophilicity of terpenoids and their saponins.  For the desired modification, glycosylation acts as an important modification strategy to improve the druggability by increasing their solubility and bioavailability [14, 15]. Glycyrrhizin (GL), a major class of terpenoids found in licorice with immuno‐regulatory, anti‐oxidative, contains two molecules of glucuronic acid (GlcA) at 3‐OH of skeleton of glycyrrhetinic acid (GA). Xu et al. reported that a glycoside hydrolase TpGUS79A, discovered from the filamentous fungus *Talaromyces pinophilus Li‐93*, can biotransform GL into glycyrrhetinic acid‐3‐O‐mono‐β‐D‐glucuronide (GAMG) by cleaving the distal GlcA. Fed‐batch fermentation in a 150‐liter fermenter was realized, so that the conversion rate of GL reached 95.3%, and the yield of GAMG was 25.3 g/L compared with GL, GAMG shows more pleasant sweetness, better safety and higher bioavailability and solubility, which will reduce the high viscosity of reaction media, and little energy was consumed. [16]. GA, the skeleton of terpenoids GL or GAMG, is the hydrolyzed aglycone of GL or GAMG by removing GlcA entirely. Wang et al. reported that a glycoside hydrolase named *At*GUS from *Aspergillus. terreus Li‐20*, that could transform GL or GAMG into GA. Considering the low water solubility, GA could be subsequently separated and purification by simple centrifugation or membrane filtration. Similarly, the hydrophilicity of the product can be increased by adding glycosides to the pentacyclic triterpene glycyrrhetinic acid [17].

### 2.2 Improve precursor supply and modification of chassis strains

Improving the supply of precursors and the modification of chassis strains can often synergistically promote the later separation of products. For example, α‐amyrin, a plant‐derived high‐value triterpene, has the effects of anti‐tumor, anti‐inflammatory, anti‐oxidant, enhancing immunity and protecting the liver. At present, it is mainly obtained by direct extraction from plants or chemical synthesis, but industrial production is restricted due to low yield, unfriendly environment and by‐products produced in the production process. Yu et al. constructed engineered *Saccharomyces cerevisiae* by modifying α‐amyrin synthase and expanding the storage pool, which made the yield of α‐Amyrin 106‐fold higher than the original strain. The key genes of the MVA pathway are overexpressed to provide sufficient precursors, while *DGA1* (diacylglycerol acyltransferase) is overexpressed to expand the intracellular storage capacity. Finally, the highest 1.1 g/L α‐amyrin among yeasts reported so far was produced through fed‐batch fermentation [18].

With the continuous development of synthetic biology and bioinformatics, metabolic pathways from different sources have been gradually elucidated. Through ingenious design, synthetic biologists modify specific metabolic pathways to greatly broaden the scope of microbial production of high value‐added products. By modifying the chassis strain, the yield of terpenoids can be increased and the downstream processing process can be simplified. Non‐oily *Saccharomyces cerevisiae* exhibits low production capacity for lipophilic natural products, especially compounds accumulated in cells, such as carotenoids. Ma et al. introduced a method for lipid engineering to promote the overproduction of lycopene in non‐oily organisms by overexpressing key genes related to fatty acid synthesis and triacylglycerol (TAG) production such as fatty acid desaturase (*OLE1*), and deleted seipin (*FLD1*) gene to adjust the size of lipid droplets. In the end, combined with the orientation of substrate transfer, the consumption of glucose is reduced, and the yield of lycopene reaches 2.37 g/L [19].

Besides, more and more specific transporters have been discovered and identified, which also promotes the production or separation of specific products. Taking the biosynthesis of the anti‐inflammatory drug hydrocortisone as an example, Over‐expression of the steroid transporter *ClCDR4* gene from *Cochliobolus lunatus*, the final hydrocortisone reached 1.06 g/L [20]. Fu et al. identified AaPDR3 as a transporter that encodes pleiotropic drug resistance. Compared with the control group, when AaPDR3 is overexpressed in yeast, the accumulation of β‐caryophyllene (a sesquiterpene) will accelerate [21]. Zhong et al. reveal the accumulation and membrane transport in *Coptis deltoides* using transcriptome analysis, and found that nine of these transporters are highly homologous to the transcripts of known alkaloid transporters, which provides the possibility for subsequent cell factory construction [22]. It is believed that more transporters will be discovered and applied to the modification of strains in the future.

### 2.3 Cell‐free system and inducible cell lysis systems

The cell‐free system is a synthetic biology method without require living cells. Products with biological functions can be synthesized in vitro, which is also a feasible and convenient method for later separation. For example, Korman et al. designed and used 27 enzymes as a system to convert glucose into different monoterpenes such as limonene, pinene and sabinene using cell‐free system. Finally, the conversion rate of pinene is greater than 95% and a titer of 15 g/L was obtained, and the system can maintain continuous operation for at least 5 days [23]. Due to the effective cell‐free system, operations such as centrifugation or cell fragmentation can be consequently reduced, which facilitates the continuity of production and reduces the number of downstream separation stages.

In recent studies, the form of promoting downstream processing is gradually applied from the perspective of upstream strain construction, but some existing methods still need to be improved and united. For example, an inducible cell self‐lysis system could release intracellular products under specific conditions such as temperature, quorum regulating system and light‐activated [24]. Hajnal et al. reported an induced lysis method based on lambda phage *SRRz* gene and synthetic ribosome binding site (RBS), which can work in *Escherichia coli* and *Halomonas campaniensis*. The production strain can be induced to lyse after adding a small amount (1‐5%) of solvent, or spontaneously lyse during downstream processing, so it is possible to eliminate mechanical cell destruction steps, improve efficiency and reduce production costs [25]. In the future, with the development of synthetic biology and bioseparation engineering, this complementary form will be more closely combined and be skillfully applied to the production of terpenoids.

## 3 COMMON SEPARATION METHODS

After microbial fermentation, the target product needs to be separated and purified from the culture medium, which is a downstream processing process. Due to the various types of terpenoid products and the complex composition of the medium, there are also various methods of separation and purification. Here, we mainly describe the properties and added value of different terpenoids from the perspective of unit separation technology, and Table 3 summarized the most commonly separation methods in various types of terpenoid purification.

**TABLE 3:** Separation and purification strategies of different terpenoid products

Product	Classification	Reaction system	Separation methods	Result	References
Limonene‐1,2‐diol	Monoterpene	*Colletotrichum nymphaea CBMAI 0864*	Four‐step extraction, Column Chromatography	Limonene‐1,2‐diol with 99% purity was obtained	[27]
Carvone	Monoterpene	*Rhodococcus erythropolis DCL14*	Solid–Liquid Two‐Phase Partitioning Bioreactor, extraction	The amount of substrate that can be added has increased by more than 7 times	[28]
Citronellol	Monoterpene	*Baker's yeast*	Distillation, multistage bioreactor	In situ removal of products without the use of solid biocatalysts and reduction of downstream processes	[60]
Citronellol	Monoterpene	*Saccharomyces cerevisiae*	Continuous‐closed‐gas‐loop bioreactor(CCGLB)	Achieve in‐situ product removal, reduce many downstream processes, and reduce overall production costs	[61]
(2R,5R)‐dihydrocarvone	Monoterpene	*Escherichia coli*	Ionic liquid or hydrophobic adsorption resin	(2R,5R)‐dihydrocarvone was obtained with 96.5% de and 96.8% conversion within 9 h	[62]
(−)‐menthol ester	Monoterpene	*Candida rugosa lipase type VII*	Thermal separation, vacuum distillation	The purity of the obtained product is as high as 94%, which is increased to 98.7% after further esterification	[63]
Perillic acid	Monoterpene	*Pseudomonas putida DSM 12264*	Anion exchange resin	The 7‐day accumulation concentration of the product reaches 31 g/L, the purity exceeds 93%, and the product loss is 2%	[64]
Eight new sesquiterpene methyl cyperenoate derivatives	Sesquiterpene	*Cunninghamella elegans AS 3.2028*	Extraction, percolating, silica gel column, crystallization, RP‐C18 silica gel column chromatography, preparative HPLC	Compounds 1–8′s yield: 8.6 mg, 6.2 mg, 5.4 mg, 7.7 mg, 9.1 mg, 5.8 mg, 6.9 mg, 4.5 mg	[65]
(–)‐(3R)‐3‐hydroxy‐isolongifolol and (–)‐(9R)‐9‐hydroxy‐isolongifolol	Sesquiterpene	*Glomerella cingulata*	Filtration, salt out, extraction, drying, evaporation, chromatographed on silica‐300 columns	Recover 420 and 102 mg, respectively	[66]
10S,14‐epoxycurcumol	Sesquiterpene	*Cunninghamella elegans AS 3.2028*	Filtration, extraction, vacuum concentration, silica gel column	The purity of the products was greater than 98% by HPLC analysis	[67]
3β‐hydroxyartemisinic acid and 3β,15‐dihydroxyartemisinic	Sesquiterpene	*Trichothecium roseum*	Extraction, drying, reduction vaporization, silica gel column, crystallization, HPLC	The former with 51.1% (204.6 mg) yield and 97.2% HPLC purity; The latter with 37.3% (149.2 mg) yield and 98.7% HPLC purity	[68]
Five new derivatives of Ladane diterpene	Diterpene	Five filamentous fungi	Solid‐phase extraction, vacuum evaporation, HPLC‐CAD, C‐18 semipreparative Kromasil column	The production volume is 6.5 to 39.9 mg/L, and the purity of each isolated compound is higher than 98%	[69]
16‐hydroxy‐mulin‐11,13‐dien‐20‐oic acid	Diterpene	*Aspergillus alliaceus*	Filtration, extraction, drying, vacuum evaporation, FCC	Produce 19.5 mg (19.5%) of biotransformation product	[70]
Nor‐annonalide	Diterpene	*Fusarium oxysporum*	Filtration, solid‐phase extraction, semi‐preparative HPLC	Crude products from the biotransformation experiments were purified by semi‐preparative HPLC and The product was isolated in up to 88.6% yield	[71]
Rebaudioside‐A	Diterpene	Glucosyl‐transferases	Multi‐stage membrane filtration, multi‐column chromatography, concentration, vacuum drying	Developed a new in situ enzymatic glycosylation method of steviol glycosides; finally obtained rebaudioside A with a purity of 95%	[33]
GAMG	Triterpene	β‐glucuronidase	Membrane filtration	The conversion rate is 33%, and the cost of separating enzymes is reduced to 1% of the original	[72]
GAMG	Triterpene	*Penicillium purpurogenum Li‐3*	The biphasic system, extraction	GAMG was produced at a higher yield in the biphasic system (87.63%). The product GAMG and the byproduct GA spontaneously separated in the biphasic system; thus, circumventing additional complex extraction processes.	[73]
GA‐3‐O‐monoglucose	Triterpene	Glycosyl‐transferase UGT73C11	Extraction, evaporation, freeze‐drying, vacuum concentration, semipreparation LC10AR	98% of GA was converted into the corresponding GA‐3‐O‐monoglucose under optimized conditions at 6 h	[74]
Oleanonic acid methyl ester	Triterpene	*Nocardia iowensis*	Centrifugation, filtration, vacuum concentration	An immobilization of cells could result in a higher productivity. As a result of the reduction of downstream steps the process becomes more efficient and economic	[75]
Betulinic acid	Triterpene	*M. canis and T. tonsurans*	Extraction, multi‐column chromatography	The purification yield was 76.92%	[76]
Betulone	Triterpene	*Dothideomycete sp. HQ 316564*	Extraction, drying, evaporation, vacuum concentration, recrystallization	The yield of betulone reached 43.4%	[77]
Panax notoginseng saponins Rd and Rg3	Triterpene	*Aspergillus niger 3.3883 and Aspergillus oryzae 3.591*	Filtration, Ultrasonic extraction, evaporation	The yields of Rd and Rg3 reached 41.40 and 6.29 mg/g which increased 6.81‐fold and 3.27‐fold to that of untreated control one, respectively	[78]
Platycodin D	Triterpene	Cellulase	High‐speed countercurrent chromatography	Approximately 39.4 mg of platycodin D (99.8% purity) was obtained from 200 mg of the product in a one‐step separation.	[35]
Betulone	Triterpene	*Rhodotorula mucilaginosa*	Extraction, concentration, preparative HPLC	35 mg betulone (purity 99.0%)	[79]
Ginsenoside Rd	Triterpene	*Cladosporium fulvum*	Filtration, extraction, vacuum evaporation, preparative HPLC	The bioconversion yield rate is 86%, and the preparation scale conversion rate is 80%	[80]
Protopanaxatriol	Triterpene	Recombinant Bacterial β‐glucosidase	Centrifugalization, extraction, Filtration, evaporation, silica column chromatography	5.45 g 20(S)‐PPTA was produced from 36 g PPT type ginsenoside mixture with 95% purity by chromatography.	[81]
Ginsenosides Rb1, Rd, F2, and compound K	Triterpene	*Paecilomyces bainier*	Extraction, reversed‐phase HPLC	The recoveries of ginsenosides were in the range of 94.4‐103.1%	[82]
Genipin	Triterpene	*Trichoderma harzianum CGMCC 2979*	Centrifugalization, XAD‐16N‐resin, concentration, silica‐gel chromatography	The total recovery rate of genipin is 62.3%	[83]
Ginsenoside Rh2	Triterpene	*Esteya vermicola CNU 120806*	Extraction, vacuum evaporation	The conversion rate is 90.7%; the target product only exists in the mycelium, and the substrate remains in the liquid layer, which is conducive to product purification and substrate recovery	[84]
Ginsenoside Rg3(S)	Triterpene	Combinative use of two glycoside hydrolases (BglBX10 and Abf22‐3)	Filtration, vacuum evaporation, column chromatography, HP20 resin	78 ± 1.2% chromatographic purity	[85]
Ginsenoside Rd	Triterpene	*Paecilomyces bainier 229‐7*	Macroporous resin	Ginsenoside Rd was purified from the culture medium by a macroporous resin with a chromatographic purity of 92.6%.	[86]
Ginsenoside CK and F1	Triterpene	A novel β‐glucosidase (MT619) from *Microbacterium testaceum A TCC 15829*	Macroporous resins	1.38 and 1.59 g of CK and F1 products were obtained, with chromatographic purity of 51.6% and 25.2%	[87]
Ginsenoside)S(‐Rg2 and)S(‐Rh1	Triterpene	A new ginsenoside‐transforming β‐glucosidase (BglQM) from *Mucilaginibacter sp. Strain QM49*	Extraction, vacuum evaporation, Biotage SNAP flash chromatography, silica column	Treated with BglQM, followed by silica column purification, to produce(S)‐Rh1 and (S)‐Rg2 at chromatographic purities of 98% ±0.5% and 97% ±1.2%, respectively.	[88]
Mogroside IV, mogroside III E, mogroside II A	Triterpene	*Saccharomyces cerevisiae*	Reversed‐phase C‐18 solid‐phase‐extraction cartridges, HPLC‐DAD‐ESI‐MS	The purities of the isolated MG‐IV, MG‐III E, and MG‐II A were 98.01%, 98.96%, and 80.54%, respectively, based on peak area in each HPLC chromatogram.	[89]

### 3.1 Extraction

Extraction is an important unit operation in the bioindustry to extract products from mixtures. It has the advantages of good selectivity, mild conditions, low energy consumption and high coupling [26]. For those terpenoids that are easily separated by properties such as polarity or solubility, we will choose relatively single separation techniques like extraction and crystallization. Generally, it can be divided into liquid‐solid extraction and liquid‐liquid extraction, which are used to extract intracellular and extracellular active ingredients, respectively. Taking multi‐stage extraction and separation of monoterpenes as an example, Medeiros et al. [27] have reported a liquid‐liquid extraction method for separating limonene‐1,2‐diol in fungal biotransformation media containing limonene as the main undesirable product. After media filtration, the supernatant was extracted in four steps by using two different solvents (hexane and ethyl acetate). The separation purity achieved was as high as 90%. In addition, they also developed a purification system using column chromatography to further obtain limonene‐1,2‐diol with a purity of 99%. In other words, the use of multi‐stage extraction can make the product reach a higher purity, reduce the follow‐up process, thereby reducing the cost of downstream processing.

With the rapid development of industrial biotechnology, many new extraction technologies have emerged in recent years, such as supercritical extraction, liquid membrane extraction, reversed micelle extraction, and aqueous two‐phase extraction. The bioconversion of carvacrol to carvone in a two‐phase partition bioreactor (TPPB) by using a type of polymer beads (Hytrel1 8206). The carvone product is successfully recovered quantitatively from the polymer, and the beads can be reused. Compared with a single aqueous phase reference reactor, the amount of substrate that can be added during the entire bioconversion process has increased by more than 7 times, which provides a method for the separation of terpenoids with large production capacity, convenient continuous operation, and even easy realization of intelligent control [28].

### 3.2 Crystallization

Crystallization, an important chemical unit operation, is widely used and effective in the biological separation of terpenoids, because it allows obtaining such target compound in a pure form and solid consistency from relatively impure solutions by a single step. During the converted separation of ginsenosides, in order to efficiently produce high‐bioactive minor ginsensoide C‐K or F2, Xiao et al. isolated a *Aspergillus niger g.848* strain consisting of ginsenosides typ1‐I, which converted PPD‐ginsenosides into C‐K. After fermentation, vacuum concentration, macroporous resin adsorption and ethanol elution, the raw product C‐K is obtained, and then recrystallized with methanol, the purity of the product rises from 85 to 98% [29]. In order to meet the requirements of these industrial productions, in the past 40 years, researchers have made a lot of efforts in the research and development of crystallization technology. Many industrial crystallization devices have also appeared in practical applications, such as vacuum crystallizers, FC continuous crystallizer and DTB crystallizer, etc.

### 3.3 Membrane filtration

Membrane separation is a multi‐disciplinary technology requires simple, less energy, and mild operating conditions that plays an important role in the plant extracts and recovery of fermentation metabolites [30]. In terpenoids separation or purification, membrane is often applied to terpenoids with high added value and difficult separation, especially those terpenoids that are difficult to separate through simple extraction and crystallization. Depending on the physicochemical composition of membrane such as charge, pore size, surface topography or hydrophilicity/hydrophobicity, even the integrated function of separation processes, the most employed for the recovery of terpenoids include microfiltration(MF), ultrafiltration(UF), nanofiltration(NF), reverse osmosis, electrodialysis, and emulsification separation [31, 32]. The essence of them is to use different substances to pass through the membrane with different transfer rates to achieve separation. Taking the membrane separation of a diterpene as an example, Adari et al. developed a new in situ enzymatic glycosylation method of steviol glycosides, which increased the content of the product rebaudioside A from 4 to 66%. In terms of product separation, the authors first purified the rebaudioside‐A present in the crude extract through multi‐stage membrane filtration (MF,UF,NF) and multi‐column chromatography, then further concentrated and dried/spray dried under reduced pressure, and finally obtained rebaudioside‐A with a purity of 95% [33].

For industrial applications, due to the complexity of the structure of terpenoids and their saponins derivatives in the separation process, it is necessary to develop new materials to increase the loading capacity of enzymes and their own separation performance, while paying attention to combine the separation of membrane separation technology with other separation technologies, and which has attracted increasing interest in the fields of bioseparation engineering, polymer materials, and physical chemistry in recent years.

### 3.4 Enzyme immobilization

In the process of biological separation, membrane separation is often combined with enzyme immobilization technology to separate terpenoids with high difficulty and added value. GAMG is a derivative of GL, which has anti‐inflammatory, anti‐allergic, anti‐cancer and anti‐viral pharmacological activities, and is also a safe new high‐density sweetener [16]. Since GAMG is amphiphilic compound, it is difficult to separate GAMG by simple extraction or recrystallization. Wu et al. developed a “smart” biological reaction system using the special characteristics of temperature‐responsive polyurethane (TRPU), combining solvent evaporation and wet‐phase conversion technology. The β‐glucuronidase was firmly fixed to the asymmetric TRPU at multiple points through biotin and streptavidin, and the asymmetric TRPU membrane was used as antennae and actuator, and the actuator was reversible to temperature responsively to switch (open or close) the channel of the reactant GL, and successfully controlled the entry of the reactant GL toward β‐glucuronidase. The bioreactor, which immobilized β‐glucuronidase on special functional membrane materials, resulted in a final GAMG yield of 33% and reduced the cost of the enzyme isolate to 1%. [34].

### 3.5 Other separation technologies

In addition to the common unit operations mentioned above, many different downstream technologies have been applied to the separation and purification of terpenoids, such as chromatography, distillation and ion exchange. For instance, chromatography technology has made outstanding contributions to the separation of plant terpenoids. Whether it is analytical chromatography or preparative chromatography, there are countless application examples. For the chromatographic separation of plant terpenoids, the more commonly used methods are high‐speed countercurrent chromatography (HSCCC) in partition chromatography and ion exchange resin in ion exchange chromatography. Among them, high‐speed countercurrent chromatography is particularly suitable for the separation of natural biologically active ingredients, which can avoid sample loss due to irreversible adsorption, so it is widely used in natural products, biomedicine and food industries. Hai et al. have developed and used a preparative HSCCC to separate and purify platycodin D from enzymatically converted saponin. By using a two‐phase solvent system composed of ethyl acetate/n‐butanol/water (1.2:1.2:1.2, v/v/v), the product after enzymatic conversion can be separated and purified in one step, and finally platycodin D with a purity of 99.8% is obtained [35]. In ion exchange chromatography, ion exchange resin is used as a stationary phase, and the fixed ionic group on the resin is reversibly transformed with the mobile phase, and separation is achieved according to the difference in affinity between them.

Besides, we take distillation as an example, such as using the different boiling points of the substrate and the product to purify the deep eutectic solvents (DESs) reaction mixture by thermal separation. After the complete conversion of the limit substrate DDA, (−)‐menthol and (−)‐menthyl dodecanoate were separated by vacuum distillation, and the purity of the obtained product was as high as 94%. After the second esterification reaction was performed on the recovered pure (−)‐menthol, the conversion rate was further increased to 98.7%.

## 4 PROSPECTS FOR COUPLING INTELLIGENT CONTROL AND MICROBIAL CELL FACTORIES WITH DOWNSTREAM PROCESSES

Undeniably, the continuous development and improvement of new unit operations are very beneficial for the separation of terpenoids. Combination with the introduction of intelligent control systems and intelligent microbial cell factories, new ideas have been brought to the downstream process. By coupling the intelligent microbial cell factory and control with downstream processes, the regulation of macro and micro production parameters can be achieved, which will further promote the production and cost reduction of terpenoids.

The parameters of the fermentation process include pH, temperature, pressure, dissolved oxygen and defoamer et al needs be monitored and automatically adjust to the production of terpenoid along with the overall system. At present, the intelligent control systems mainly used in biochemical enterprises include distributed control systems (DCS) and programmable logic controller (PLC). Compared with PLC, the DCS can be comprehensively applied to the entire production process. The main feature is centralized management and decentralized control. The cost of the system is higher and maintenance is more difficult. However, PLC mainly focuses on unitized intelligent control and monitoring, such as monitoring and automatic adjustment of parameters such as pH, pressure, dissolved oxygen, and defoamer in the fermentation process. Thanks to the development of intelligent control, we can get real‐time feedback on the production status provided by these control systems from the computer. In addition to obtain the best effect of operation, DCS and PLC can also set multiple parameters according to the actual operating conditions of the user. Obviously, this also provides a global control for the separation and purification of biological products, especially terpenoids.

In recent years, the trend of integrating intelligent microbial cell factories, intelligent control and separation into integrated processes has attracted more and more attention. In order to realize the automatic control of certain culture conditions from the production source and reduce the difficulty and cost of downstream separation, the intelligent cell factory has become a research hotspot in synthetic biology and bioseparation engineering. In industrial fermentation, microbial metabolism usually causes pH changes, which affect the performance of strains and the supply of downstream precursors. The commonly used method to control the pH value is to add acid or alkali after the control system detects the pH value change, which usually increases the cost and complexity of downstream separation. In order to achieve self‐regulation of pH, Li et al. designed and constructed “Genetic pH Shooting” (GPS) consisting of a pH‐sensing promoter and acid/alkali‐generating genes. During the large‐scale fermentation process, the lycopene titer in the engineered strains carrying GPS increased by 137.3%, and the ammonia usage decreased by 35.6% [36]. Similarly, temperature is another important factor affecting the production efficiency of engineered strains. Jia et al. developed an intelligent microbial heat regulation engine (IMHeRE) to improve the heat resistance of *E. coli*. At the cellular level, a heat‐resistant system composed of different heat shock proteins and RNA thermometers gradually expands the optimal temperature by sensing thermal changes. In practical applications, the production of lysine at 40°C is increased by five‐fold when IMHeRE is used [37].

Besides, combining intelligent cell factories with two‐stage fermentation has become a viable idea for increasing production. In order to further resolve the contradiction between cell growth and tocotrienol accumulation to achieve high‐density fermentation, the researchers designed a cold shock‐triggered temperature control system to effectively control the two‐stage fermentation, resulting in 320 mg/L tocotrienols [38]. In addition, based on the comprehensive engineering of subcellular compartments, the dual metabolic engineering of cytoplasm and mitochondrial acetyl‐CoA is used to increase the synthesis of isoprene in *S. cerevisiae*. This strategy increased the production of isoprene by 2.1‐fold and 1.6‐fold. In the two‐stage culture process, dynamic metabolic regulation and aerobic fed‐batch fermentation are carried out to fully provide acetyl‐CoA and carbon, and finally 2.527 g/L of isoprene is obtained [39].

## 5 CONCLUDING REMARKS

With the continuous emergence and development of new separation technologies, various separation technologies have been successfully applied to actual production and this is confirmed. However, for the separation and purification of terpenoids such as amphiphilic and thermally unstable terpenoids, there are still certain challenges. Simultaneously, different unit separation operations usually have certain limitations. For instance, certain extraction techniques may require the use of a large amount of organic reagents, or the membranes in the membrane separation process may be easily fouled and reduce membrane performance. Undoubtedly, single or simple separation technology is bound to be difficult to meet the demand. Therefore, according to the physical and chemical properties of terpenoids and the composition of the fermentation broth, combining different unit separation operations to form a targeted integrated separation process, even an intelligent control process, will be the focus of future research in the field of plant terpenoid separation.

## CONFLICT OF INTEREST

The authors have declared no conflict of interest.

## Data Availability

All data, models, and code generated or used during the study appear in the submitted article.
